# Vascular Smooth Muscle‐Secreted Exosomal X26nt Impedes Atherosclerosis Progression via the c‐FOS/XBP1/SOD1 Axis

**DOI:** 10.1002/iid3.70251

**Published:** 2025-08-26

**Authors:** Zhibin Zhang, Dachang Liu, Yue Zheng, Yanwu Liu, Xian Cheng, Yun Chang, Xiaoyu Liang, Xiaomin Hu, Wenqing Gao

**Affiliations:** ^1^ School of Medicine Nankai University Tianjin China; ^2^ Department of Heart Center The Third Central Hospital of Tianjin; Nankai University Affiliated Third Center Hospital Tianjin China; ^3^ Department of Heart Center Tianjin Key Laboratory of Extracorporeal Life Support for Critical Diseases Tianjin China; ^4^ Department of Heart Center Tianjin ECMO Treatment and Training Base Tianjin China; ^5^ Department of Heart Center Tianjin Medical University Tianjin China

**Keywords:** autocrine effects, IRE1a, SOD1, VSMCs, X26nt, XBP1

## Abstract

**Background:**

Atherosclerosis is a chronic immune‐inflammatory disorder in which vascular smooth muscle cell (VSMC) phenotypic modulation plays a critical role in plaque development and instability. Endoplasmic reticulum (ER) stress and its downstream effector, XBP1s, have been shown to influence VSMC behavior. During XBP1 mRNA splicing, a 26‐nucleotide RNA fragment (X26nt) is excised, yet its biological significance remains poorly understood. Exosomes derived from VSMCs have been implicated in mediating intercellular signaling under inflammatory and stress conditions. However, the potential role of X26nt in vascular regulation, particularly via exosomal pathways, has not been investigated.

**Methods:**

Atherosclerosis was induced in ApoE‐/‐ mice using a high‐fat diet. Ox‐LDL‐treated VSMCs were used for in vitro studies. Histology, qPCR, and Western blot were conducted. Exosomes from IRE1α‐ or XBP1‐knockdown VSMCs were isolated and used to treat Ox‐LDL‐exposed VSMCs to assess X26nt effects. Luciferase assays and ChIP were used to explore mechanisms. AAV2‐SM22a‐ZsGreen‐26nt vectors were constructed to evaluate X26nt effects in vivo.

**Results:**

X26nt levels in exosomes increased with arterial medial thickening in atherosclerosis. In vitro, exosomal X26nt decreased ER stress, suppressed mitophagy, and upregulated SOD1 in VSMCs. Exosomes from IRE1α‐ or XBP1‐knockdown VSMCs reversed the protective phenotype. Mechanistically, X26nt bound the 3'UTR of XBP1 and c‐Fos, reducing their expression. ChIP confirmed c‐Fos directly activated XBP1 transcription. In vivo, AAV2‐X26nt delivery elevated SOD1, reduced mitophagy, and attenuated vascular remodeling.

**Conclusion:**

This study identified exosomal X26nt as a novel regulator of VSMC phenotypic switching and oxidative stress through the c‐Fos/XBP1/SOD1 axis. These findings highlight the functional relevance of ER stress‐derived noncoding RNAs in vascular remodeling and suggest that targeting exosomal RNAs, such as X26nt, may represent a promising therapeutic strategy for atherosclerosis and related cardiovascular diseases.

## Graphical abstract

1. Vascular smooth muscle cells secreted exosomal X26nt in atherosclerosis. 2. X26nt decreased ER response and mitophagy and increased SOD1 expression in VSMCs. 3. X26nt regulated XBP1 transcription and translation in VSMCs.

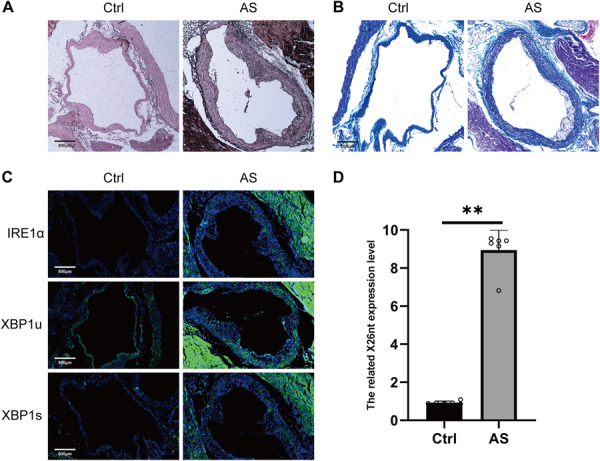

## 1 Introduction

Atherosclerosis is a chronic immune‐inflammatory disease marked by lipid accumulation, endothelial dysfunction, and immune cell infiltration into the arterial wall [1, 2]. These pathological changes drive vascular remodeling and plaque formation, ultimately leading to cardiovascular events such as myocardial infarction and stroke [3, 4]. Among the cellular contributors, vascular smooth muscle cells (VSMCs) are central to disease progression. VSMCs exhibit remarkable phenotypic plasticity, transitioning from a contractile to a synthetic phenotype, acquiring proliferative and migratory capabilities that contribute to neointimal hyperplasia and plaque instability [5, 6].

VSMC phenotypic modulation is tightly regulated by various environmental stressors, including oxidative stress and inflammation. Endoplasmic reticulum (ER) stress and the unfolded protein response (UPR) are particularly crucial in regulating vascular pathology [7, 8]. X‐box binding protein 1 (XBP1), a key transcription factor in the UPR, undergoes IRE1α‐mediated splicing to produce XBP1s, an active isoform that regulates genes involved in protein folding, redox balance, and inflammation [9, 10]. A 26‐nucleotide RNA fragment (X26nt) is excised during this splicing process. While the role of XBP1s in vascular biology is well established, the biological significance of X26nt remains largely unexplored.

Exosomes, a subclass of extracellular vesicles secreted by most cell types, have gained recognition as significant mediators of intercellular communication during inflammation and vascular remodeling [11]. These vesicles often reflect the intracellular stress status of the parent cell and are involved in regulating immune responses, oxidative stress, and phenotypic plasticity within the vascular microenvironment [12, 13, 14]. Based on this, X26nt might be released through exosomal pathways as part of a stress‐induced adaptive mechanism, contributing to vascular homeostasis.

This study showed elevated X26nt expression in the arterial wall of atherosclerotic mice, suggesting its potential involvement in disease regulation. Further investigations revealed that X26nt modulates oxidative stress and inhibits phenotypic switching in VSMCs. Mechanistically, these effects are mediated through the c‐FOS/XBP1/SOD1 signaling axis, indicating a feedback loop in ER stress regulation.

In summary, these findings highlight X26nt, a previously overlooked byproduct of XBP1 splicing, as an endogenous regulator of VSMC behavior in vascular inflammation. This study uncovers a novel mechanism linking ER stress–derived RNA fragments to immune‐inflammatory vascular remodeling and positions X26nt, potentially acting via exosomal routes, as a promising therapeutic target for atherosclerosis and related vascular disorders.

## 2 Methods

### 2.1 Cell Source and Processing

The VSMC cell line was obtained from the Chinese Academy of Medical Sciences and cultured in DMEM/F12 medium supplemented with 10% (v/v) fetal bovine serum (FBS) in a 37°C, 5% CO_2_ incubator until use in experiments.

IRE1α and XBP1s knockdown in VSMCs was performed, and exosomes were extracted using ultracentrifugation and the sucrose pad method. In brief, cells were cultured in 10 cm dishes and transfected with siRNAs targeting IRE1α and XBP1s. A total of 30 μL of Lipofectamine 2000 (11668‐019, Invitrogen) was used, with 50 nM of siRNA pools (siIRE1α pool: mmu‐IRE1α‐1, mmu‐IRE1α‐2, and mmu‐IRE1α‐3; siXBP1 pool: mmu‐XBP1‐1, mmu‐XBP1‐2, and mmu‐XBP1‐3; RIBIBIO, China) and 250 μL OptiMEM medium (31985‐070, Gibco) per dish. Nonsense sequences were used as negative controls (210011, Ubigene).

To enhance the expression of X26nt in VSMCs, X26nt was transfected using 30 μL of Lipofectamine 2000, with 100 nM X26nt (30 μL) and 250 μL OptiMEM medium per dish. To induce atherosclerosis in vitro, Ox‐LDL (25 μg/mL and 50 μg/mL, IO1300, Solarbio, China) was used to treat the cells.

### 2.2 Mice Atherosclerosis Model and Treatment

Male ApoE‐deficient mice (7–8 weeks old, weighing 22–25 g) were sourced from Charles River (Beijing, China). The mice were housed in a specific pathogen‐free environment with unrestricted access to food and water, under a 12‐h light‐dark cycle. The study protocol was approved by the Ethics Committee of Nankai University (no. 2022‐SYDWLL‐000486).

To induce atherosclerosis, ApoE‐/‐ male mice were fed a high‐fat diet (HFD, Western diet, HFHC100244) or chow diet for 2 months. To investigate the effects of X26nt on atherosclerosis, AAV2‐SM22a‐ZsGreen‐26nt and AAV2‐SM22a‐ZsGreen‐NC vectors were synthesized and used for the treatment of atherosclerotic ApoE‐/‐ male mice over 2 months (HANBio, China).

For animal welfare, mice were euthanized under deep isoflurane anesthesia (5%, MSS‐3, England) via cervical dislocation.

### 2.3 Histology Analysis

Following euthanasia, aortic samples were collected, fixed in 4% paraformaldehyde overnight, and embedded in paraffin (6 μm). Anatomical morphology changes and collagen deposition were assessed by Alizarin Red S staining (C0148S, Beyotime, China) and Masson's trichrome staining (G1340, Solarbio, China). Images were captured using a light microscope (ECLIPSE E100, Nikon, Japan).

For immunofluorescence analysis, tissue slides were incubated overnight at 4°C with the following primary antibodies: IRE1α (CST#3294 s), XBP1 (Proteintech, 25997‐1 AP), XBP1s (Proteintech, 24868‐1 AP), Osteopontin (CST#88742s), α‐SMA (Proteintech, 67735), and CD31 (Proteintech, 11265‐1‐AP). The secondary antibodies used were Alexa Fluor 488 and Alexa Fluor 594 conjugates (Abcam, both 1:200). Nuclei were stained with DAPI (Southern Biotech, USA). Images were captured using a fluorescence microscope (ECLIPSE TS2R, Nikon, Japan), and the staining areas were quantified using ImageJ software.

### 2.4 Exosome Extraction and Detection

Exosomes were isolated using differential centrifugation, as previously described [13]. In brief, to isolate exosomes from VSMCs treated with ox‐LDL, culture supernatants were collected and subjected to sequential centrifugation steps: first at 300 g for 10 min and then at 2000 g for 20 min to remove intact cells and debris. A subsequent centrifugation at 10,000 g for 30 min was used to remove large microvesicles. The raw exosomes were pelleted by ultracentrifugation at 150,000 g for 70 min. The resulting EV pellets were washed with PBS and then ultracentrifuged at 100,000 g for 70 min at 4°C. After discarding the supernatant, the exosomes were resuspended in PBS and stored at −80°C for further experiments. All procedures were performed under sterile conditions. Exosomes were characterized using transmission electron microscopy (TEM), electrophoresis, Brownian motion video analysis, laser scattering microscopy, and Western blot (WB) detection.

The primary antibodies were as follows: CD9 (1:1000, 20597‐1‐AP, Proteintech), CD63 (1:500, PA5‐92370, Invitrogen), TSG101 (1:5000, 67381‐1‐lg, Proteintech), Calnexin (10427‐2‐AP, Proteintech), and HSP70 (1:5000, 10995‐1‐AP, Proteintech).

### 2.5 qPCR Analysis

mRNA was extracted from aortic tissues and cultured cells, followed by qRT‐PCR. Total RNA was isolated using TRIzol reagent and then reverse‐transcribed using TB Green Premix Ex Taq II (TaKaRa, RR820). U6 was used as an internal reference, and gene expression was calculated using the 2^−ΔΔCt^ method. Primer details are provided in Supplementary Table.

### 2.6 Western Blot

For protein extraction, RIPA buffer (CWBIO, China) was used. Protein concentration was determined using a BCA protein assay kit. The protein samples were mixed with SDS‐PAGE loading buffer, electrophoresed by SDS‐PAGE, and transferred to a Millipore or GE PVDF membrane. The membranes were blocked with 5% skimmed milk for 1 h, followed by incubation with primary and secondary antibodies (1:5000, Affinity, China). Protein detection was performed using an enhanced chemiluminescence (ECL) system, and the results were analyzed with an image system software (Protein Simple, USA).

The primary antibodies used for WB were as follows: anti‐Parkin (1:2000, ab77924, Abcam), anti‐LC3B (1:2000, ab192890, Abcam), anti‐HMGB1 (1:10000, ab79823, Abcam), anti‐XBP1 (1:1000, ab37152, Abcam), anti‐SOD1 (1:5000, 10269‐1‐AP, Proteintech), anti‐α‐SMA (14395‐1‐AP, Proteintech), anti‐Osteopontin (22962‐1‐AP, Proteintech), and anti‐β‐actin (1:10000, EM21002, HUABIO).

### 2.7 Luciferase Experiment

To investigate the effects of X26nt on c‐FOS and XBP1 mRNA expression, the 3'UTRs of c‐FOS and XBP1 were cloned into luciferase reporter gene vectors (pPGK‐Fluc‐Fosl1(mouse)‐3'UTR‐hRluc‐Neo, pPGK‐Fluc‐Xbp1(mouse)‐3'UTR‐hRluc‐Neo). Luciferase activity was measured using a microplate reader (VICTOR Nivo Multimode Microplate Reader, PerkinElmer).

### 2.8 Chromatin Immunoprecipitation Assay

Smooth muscle cells were fixed at room temperature with formaldehyde for 10 min, followed by PBS washing. The cells were then subjected to ultrasonic treatment in a water bath (60 W, SCIENTZ‐48, SCIENTZ, China) to fragment the long strand DNA into 200–1,000 bp DNA fragments. The samples were centrifuged at 16,000 x g for 15 min at room temperature to collect the supernatant. A 20 µL aliquot of the supernatant was reserved as the input. To immunoprecipitate c‐FOS, 2.5–5 µg of c‐FOS antibody (ab222699) or the corresponding IgG antibody (A7016; Beyotime, China) was added to the remaining supernatant, followed by overnight incubation at 4°C. Magnetic beads (CST #9005) were then added, and the mixture was incubated at room temperature for 2 h. After centrifugation at 16,000 × g for 2 min at 4°C, the precipitate was eluted stepwise with low‐salt buffer, high‐salt buffer, and NaCl solution to remove chromatin fixation. The mixture was then treated with EDTA, Tris‐HCl, and protease and incubated at 65°C for 1 h. The final eluted product was purified using the phenol‐chloroform method, and 50 µL of the purified product was used for XBP1 mRNA detection. Primer details are provided in Supplementary Table 2.

### 2.9 Measurement of Mitochondrial Membrane Potential

The mitochondrial membrane potential (MMP) was evaluated using the JC‐1 assay kit (Beyotime), following the manufacturer's guidelines. Fluorescence intensity was immediately measured with a fluorescence microscope. The MMP was quantified by calculating the ratio of the green fluorescence intensity (indicative of the monomer) to the red fluorescence intensity (indicative of the polymer).

### 2.10 Analysis of Reactive Oxygen Species (ROS)

ROS generation was assessed using DCFH‐DA (Beyotime Institute of Biotechnology), according to the manufacturer's instructions. Fluorescence intensity was promptly measured using fluorescence microscopy.

### 2.11 Intracellular Calcium Measurements

Cells were seeded onto glass coverslips in a 24‐well plate and incubated overnight. Following the manufacturer's instructions, the cells were treated with 4 μM Fluo‐4 AM (Beyotime, S1061M) in DMEM, maintaining a dark environment and 37°C for 30 min. The cells were then washed twice with PBS. Fluorescence signals were captured using a fluorescence microscope (ECLIPSE TS2R, Nikon, Japan) with an excitation wavelength of 494 nm and an emission wavelength of 516 nm. The stained areas were quantified using ImageJ software.

### 2.12 Statistical Analysis

Data are presented as mean ± SD or median (Q1–Q3). Statistical analyses were conducted using SPSS 23.0. The Shapiro‐Wilk test for normality, Welch's t‐test (for two groups), and one‐ or two‐way ANOVA with Bonferroni post‐hoc tests were applied for comparisons between groups. A *P*‐value of less than 0.05 was considered statistically significant. Randomization and blinding strategies were employed whenever possible. All experiments were independently repeated at least three times, and *n* represents the number of independent biological replicates.

## 3 Results

### 3.1 Exosomal X26nt Increased When the Thickness of Medial Artery Membrane Increased in Atherosclerosis

**Figure 1:**
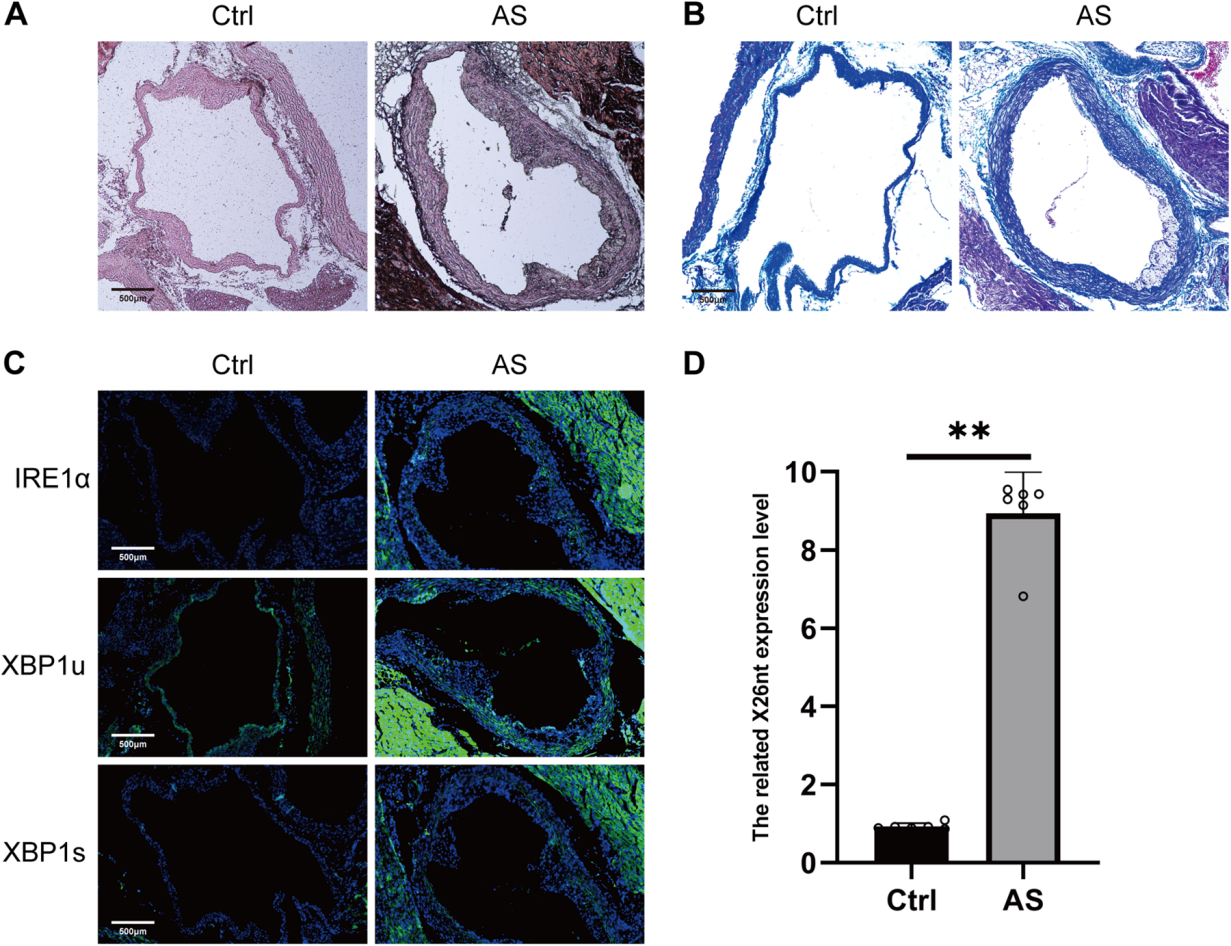
Exosomal X26nt levels increase with medial artery membrane thickening in atherosclerosis. (A‐B) Representative images of Alizarin red S staining (A) and Masson's staining (B) in aortic samples from atherosclerotic and control mice. (C) Immunofluorescent staining for IRE1α, XBP1u, and XBP1s in aortic samples from atherosclerotic and control mice. (D) qPCR analysis of X26nt in aortic samples from atherosclerotic and control mice. Mice were fed a high‐fat diet (atherosclerosis) or chow diet (control) for 2 months. *n* = 6 mice per group. Each data point represents an individual animal. Data are expressed as mean ± SD. Statistical significance was determined using Welch's *t*‐test. **p < 0.01.

To assess the effects of X26nt on medial artery membrane thickness and atherosclerosis, Alizarin Red S and Masson's trichrome staining were performed on the aortic roots of both atherosclerotic and control mice. These analyses revealed an increase in the thickness of the medial artery membrane in the atherosclerotic group (Figure 1A,B). Immunofluorescent staining of IRE1α, XBP1u, and XBP1s in aortic samples from both groups showed that IRE1α activation triggered XBP1 splicing in the medial artery of atherosclerotic mice (Figure 1C). Notably, the spliced XBP1 product, X26nt, was elevated in atherosclerosis (Figure 1D).

**Figure 2:**
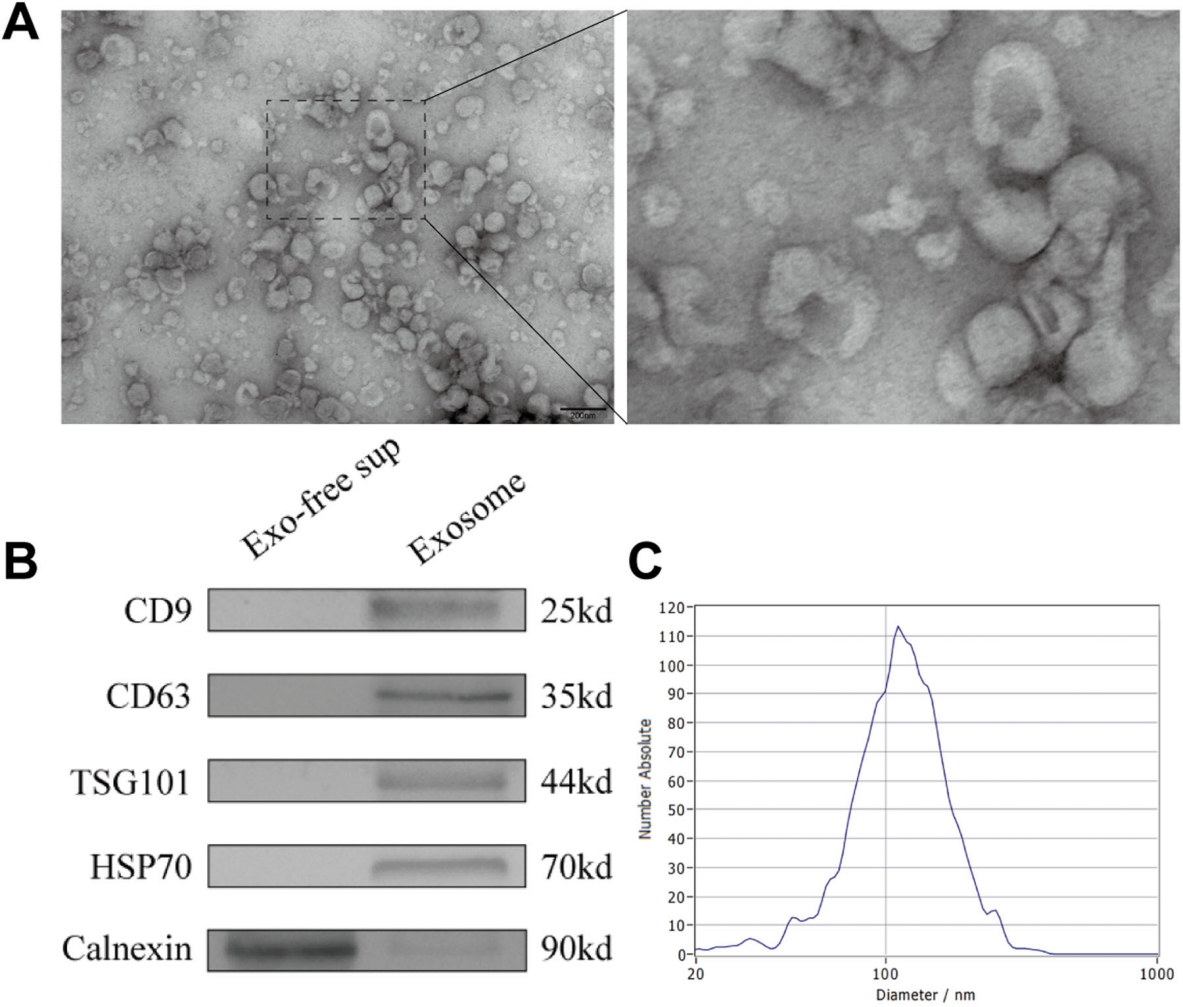
Characterization of VSMC‐derived exosomes. (A) Transmission electron microscopy image of VSMC‐derived exosomes. (B) Representative WB images showing the presence of CD9, CD63, TSG101, Calnexin, and HSP70 in exosome‐depleted supernatant and exosomes. (C) Nanoparticle tracking analysis of exosome diameter and concentration.

**Figure 3:**
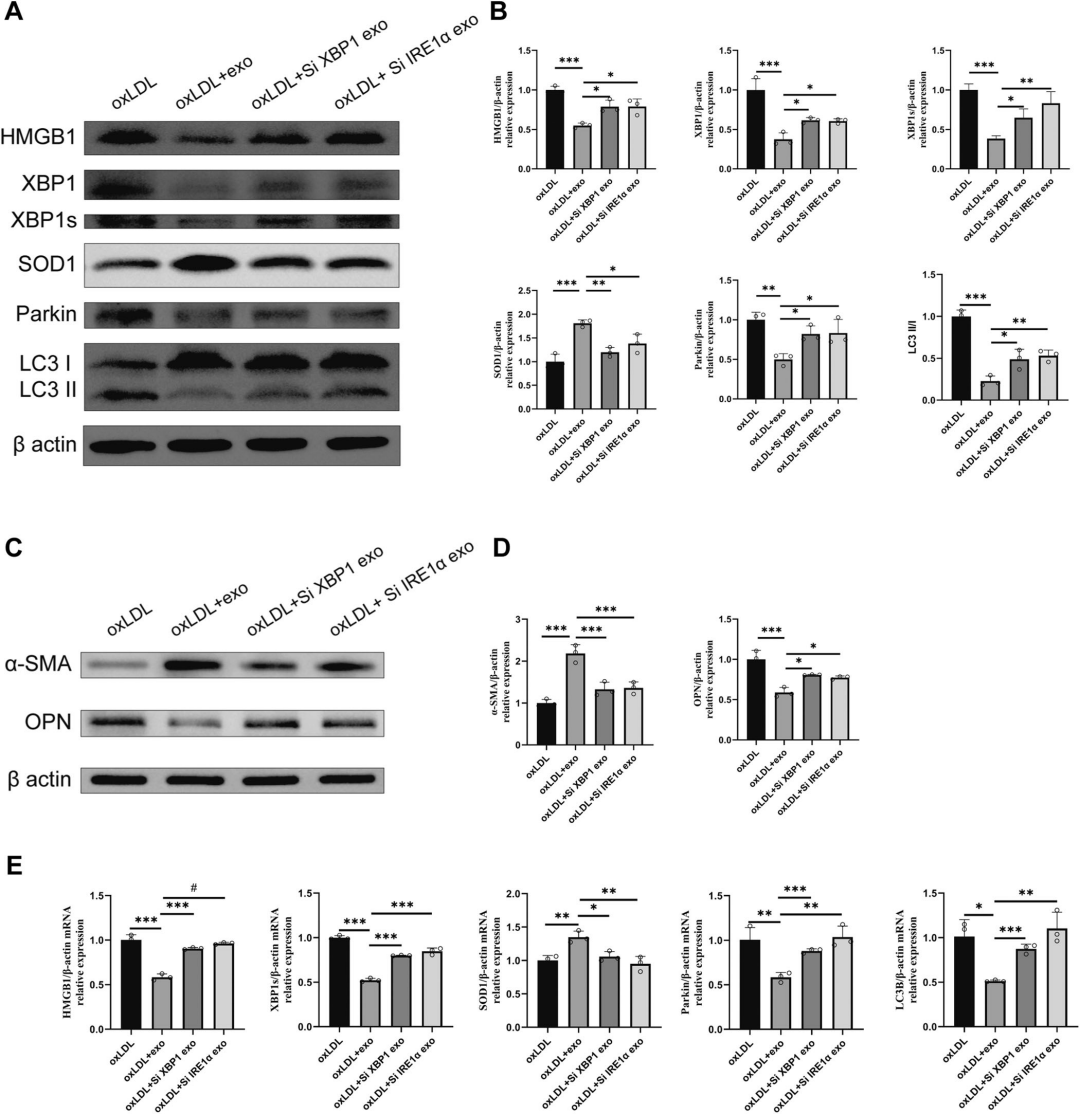
Exosomes from VSMCs reduced ER response, decreased mitophagy, and modulated VSMC phenotypic switching. (A, B) Representative WB images (A) and quantitative analysis (B) of protein levels of HMGB1, XBP1, XBP1s, SOD1, Parkin, and LC3 in VSMCs treated with exosomes from IRE1α‐knockdown, XBP1‐knockdown VSMCs, or control. (C‐D) Representative WB images (C) and quantitative analysis (D) of protein levels of α‐SMA and OPN in VSMCs treated with exosomes from IRE1α‐knockdown, XBP1‐knockdown VSMCs, or control. (E) qPCR analysis of mRNA levels of HMGB1, XBP1s, SOD1, Parkin, and LC3B in VSMCs treated with exosomes from IRE1α‐knockdown, XBP1‐knockdown VSMCs, or control. *n* = 3 per group. Data are expressed as mean ± SD. Statistical significance was determined using one‐way ANOVA and Tukey's post hoc test. *p < 0.05; **p < 0.01; ***p < 0.001; ns, not significant.

3.2 X26nt regulates autophagy and phenotypic switching in VSMCsTo investigate the autocrine effects of X26nt on VSMCs, exosomes were extracted and characterized through TEM, electrophoresis, Brownian motion video analysis, laser scattering microscopy, and WB detection (Figure 2). Exosomes derived from IRE1α‐ and XBP1‐knockdown VSMCs, or controls, were introduced to ox‐LDL‐treated VSMCs (Figure 3). Compared to ox‐LDL‐treated VSMCs, exosome treatment resulted in reduced ER response and mitophagy, while SOD1 expression was elevated. These phenotypic changes were reversed in ox‐LDL‐treated VSMCs with exosomes from IRE1α or XBP1 knockdown cells, suggesting that exosomal X26nt influences VSMC behavior. Importantly, ox‐LDL stimulation induced a phenotypic shift in VSMCs toward the synthetic state, as indicated by increased OPN and decreased α‐SMA expression (Figure 3C). This shift was partially reversed by exosomes from control VSMCs, but not by those derived from IRE1α‐ or XBP1‐silenced cells.

**Figure 4:**
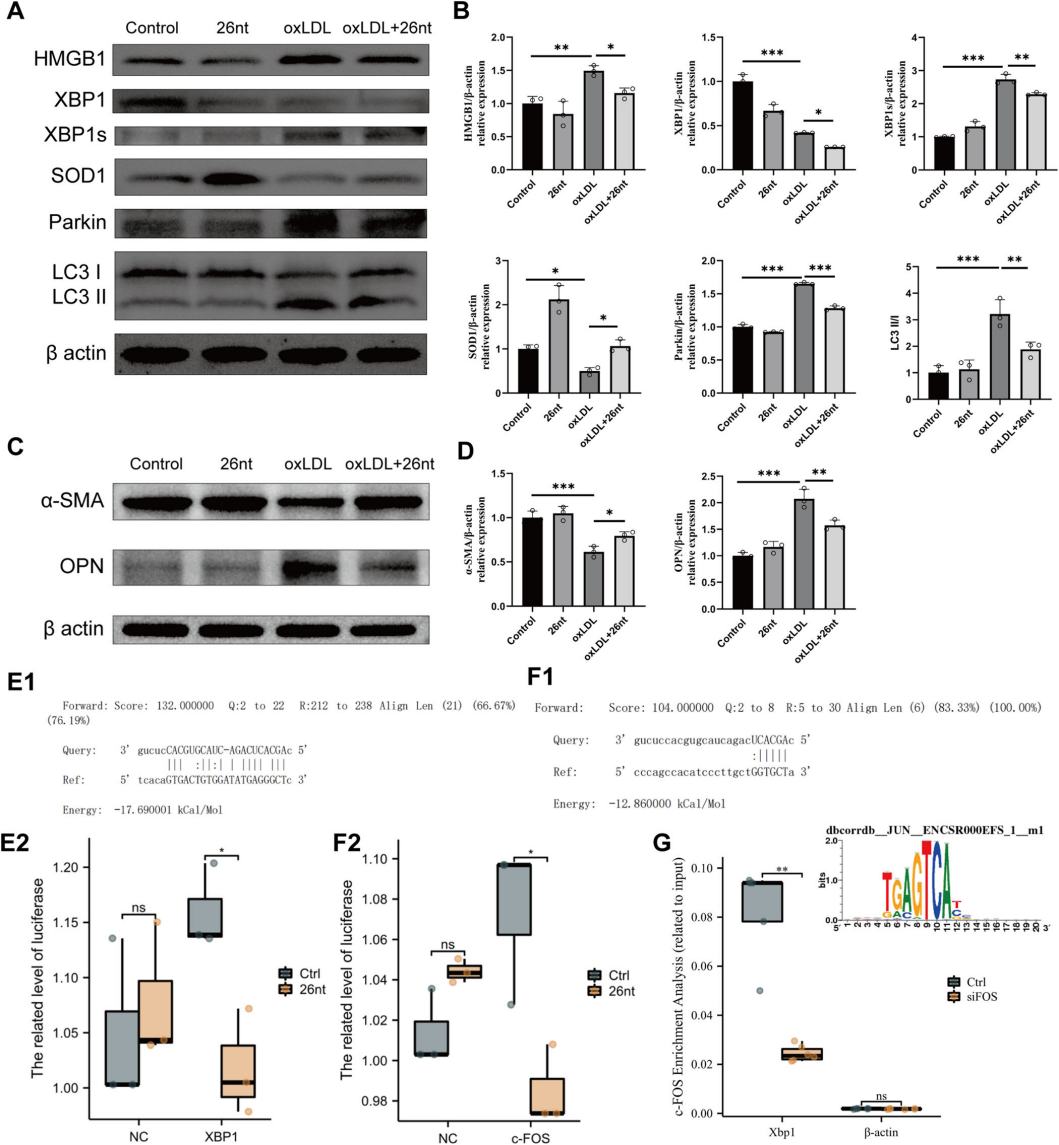
X26nt modulates XBP1 transcription and translation to increase SOD1 expression and reduce mitophagy and ER response. (A, B) Representative WB images (A) and quantitative analysis (B) of protein levels of HMGB1, XBP1, XBP1s, SOD1, Parkin, and LC3 in VSMCs treated with X26nt or control. (C, D) Representative WB images (C) and quantitative analysis (D) of protein levels of α‐SMA and OPN in VSMCs treated with X26nt or control. (E) Luciferase assay showing the effect of XBP1 and X26nt. (F) Luciferase assay showing the effect of c‐Fos and X26nt. (G) ChIP analysis and motif analysis showing c‐Fos promotes XBP1 transcription by binding the XBP1 promoter. *n* = 3 per group. Data are expressed as mean ± SD. Statistical significance was determined using one‐way ANOVA and Tukey's post hoc test. *p < 0.05; **p < 0.01; ***p < 0.001; ns, not significant.

Moreover, transfection of X26nt into ox‐LDL‐treated VSMCs led to decreased HMGB1 and XBP1 expression, alongside increased SOD1 levels, in both treated and untreated conditions (Figure 4A,B). These findings were corroborated by qRT‐PCR, which showed consistent trends in gene expression (Figure S1). Furthermore, X26nt treatment reduced mitophagy‐related protein levels in ox‐LDL‐treated VSMCs (Figure 4B). Corresponding qRT‐PCR analysis indicated that X26nt downregulated autophagy‐related genes exclusively under ox‐LDL stimulation (Figure S1).

To further evaluate the impact of X26nt on VSMC phenotypic modulation, WB was performed to measure α‐SMA and OPN expression. The results demonstrated that X26nt mitigated the ox‐LDL‐induced shift to the synthetic phenotype, as evidenced by increased α‐SMA and reduced OPN levels (Figure 4C,D).

Additionally, considering the role of endothelial dysfunction in atherosclerosis, the potential protective effect of X26nt on endothelial cells was investigated. Immunofluorescent staining showed that ox‐LDL reduced CD31 expression, while X26nt treatment partially restored this reduction, suggesting that X26nt may alleviate ox‐LDL‐induced endothelial injury (Figure S2).

### 3.2 X26nt Regulated XBP1 Transcription and Translation in Vsmcs

Luciferase assays for XBP1 and X26nt demonstrated that X26nt bound to the XBP1 3'UTR, leading to a reduction in XBP1 expression in VSMCs (Figure 4E). Similarly, luciferase assays for c‐Fos and X26nt showed that X26nt bound to the c‐Fos 3'UTR in VSMCs (Figure 4F). Notably, ChIP analysis and motif analysis revealed that c‐Fos promoted XBP1 transcription by binding to the XBP1 promoter (Figure 4G). These results indicate that X26nt regulates both the transcription and translation of XBP1 in VSMCs.

### 3.3 X26nt Increased SOD1 Expression and Decreased Mitophagy and Medial Artery Membrane Thickness in Atherosclerosis

**Figure 5:**
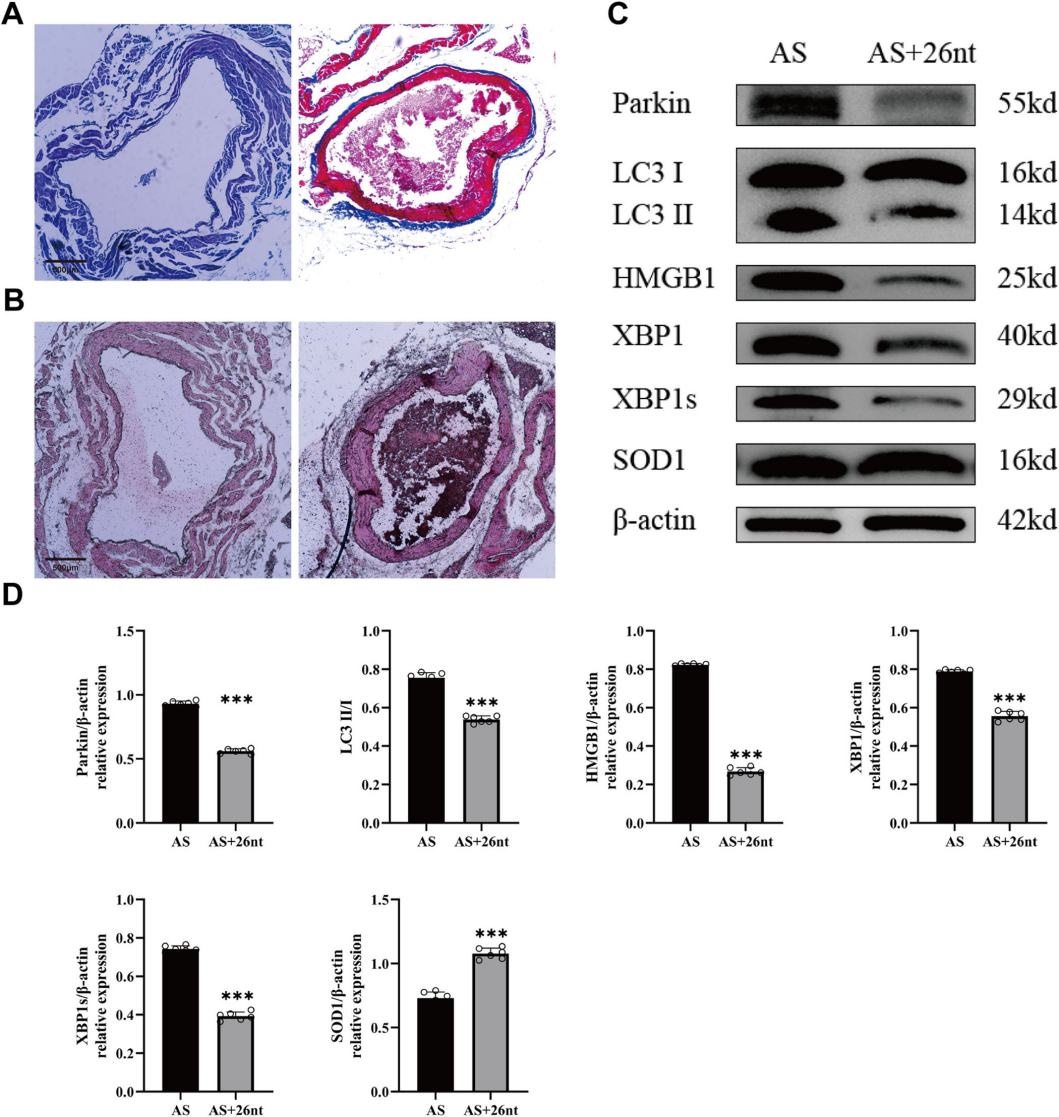
X26nt increases SOD1 expression, reduces mitophagy, and decreases medial artery membrane thickness in atherosclerosis. (A, B) Representative images of Alizarin Red S staining (A) and Masson's staining (B) in atherosclerotic mice treated with control or X26nt. (C, D) Representative WB images (C) and quantitative analysis (D) of aortic samples from atherosclerotic mice treated with control or X26nt. *n* = 6 mice per group. Each data point represents an individual animal. Data are expressed as mean ± SD. Statistical significance was determined using Welch's *t*‐test. *p < 0.05; **p < 0.01; ***p < 0.001; ns, not significant.

To examine the effects of X26nt on medial artery membrane thickness and atherosclerosis, Alizarin Red S and Masson's staining were performed on aortic roots of atherosclerotic mice treated with either control or AAV2‐SM22a‐ZsGreen‐26nt. The results demonstrated a decrease in medial artery membrane thickness in the atherosclerotic mice treated with AAV2‐SM22a‐ZsGreen‐26nt (Figure 5A,B). WB analysis showed that X26nt decreased HMGB1 and XBP1 expression, while increasing SOD1 expression in the atherosclerotic mice. Additionally, X26nt reduced mitophagy in these mice (Figure 5C,D).

### 3.4 In Vitro, X26nt Inhibits Oxidative Stress in VSMC Under oxLDL Conditions

**Figure 6:**
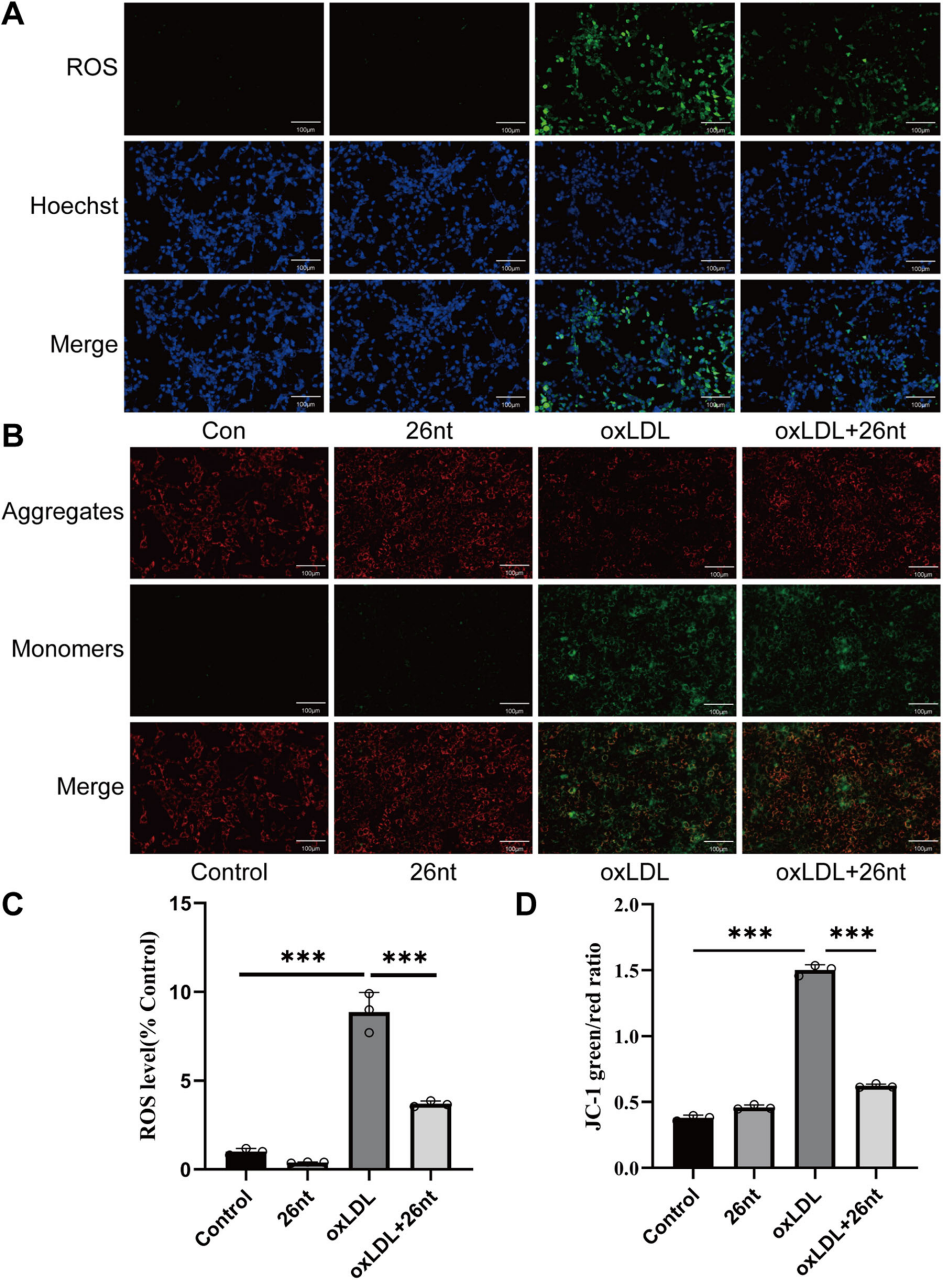
In vitro, X26nt inhibits oxidative stress in VSMCs under oxLDL conditions. (A, B) Representative images of ROS (A) and JC‐1 (B) staining in oxLDL‐treated VSMCs treated with control or X26nt. (C, D) Quantitative analysis of ROS levels and the green/red fluorescence ratio. *n* = 3 per group. Data are expressed as mean ± SD. Statistical significance was determined using one‐way ANOVA and Tukey's post hoc test. *p < 0.05; **p < 0.01; ***p < 0.001; ns, not significant.

To assess the effect of X26nt on oxidative stress in oxLDL‐treated VSMCs, ROS generation and JC‐1 staining were performed (Figure 6). OxLDL significantly induced ROS generation in VSMCs, which was partially inhibited by X26nt. Furthermore, MMP levels, an indicator of oxidative stress, showed that X26nt partially alleviated the oxLDL‐induced alterations in MMP.

### 3.5 In Vitro, X26nt Inhibits Cellular Calcium Overload Under oxLDL Conditions and Promotes the Transition of VSMC From Synthetic to Contractile Phenotype

**Figure 7:**
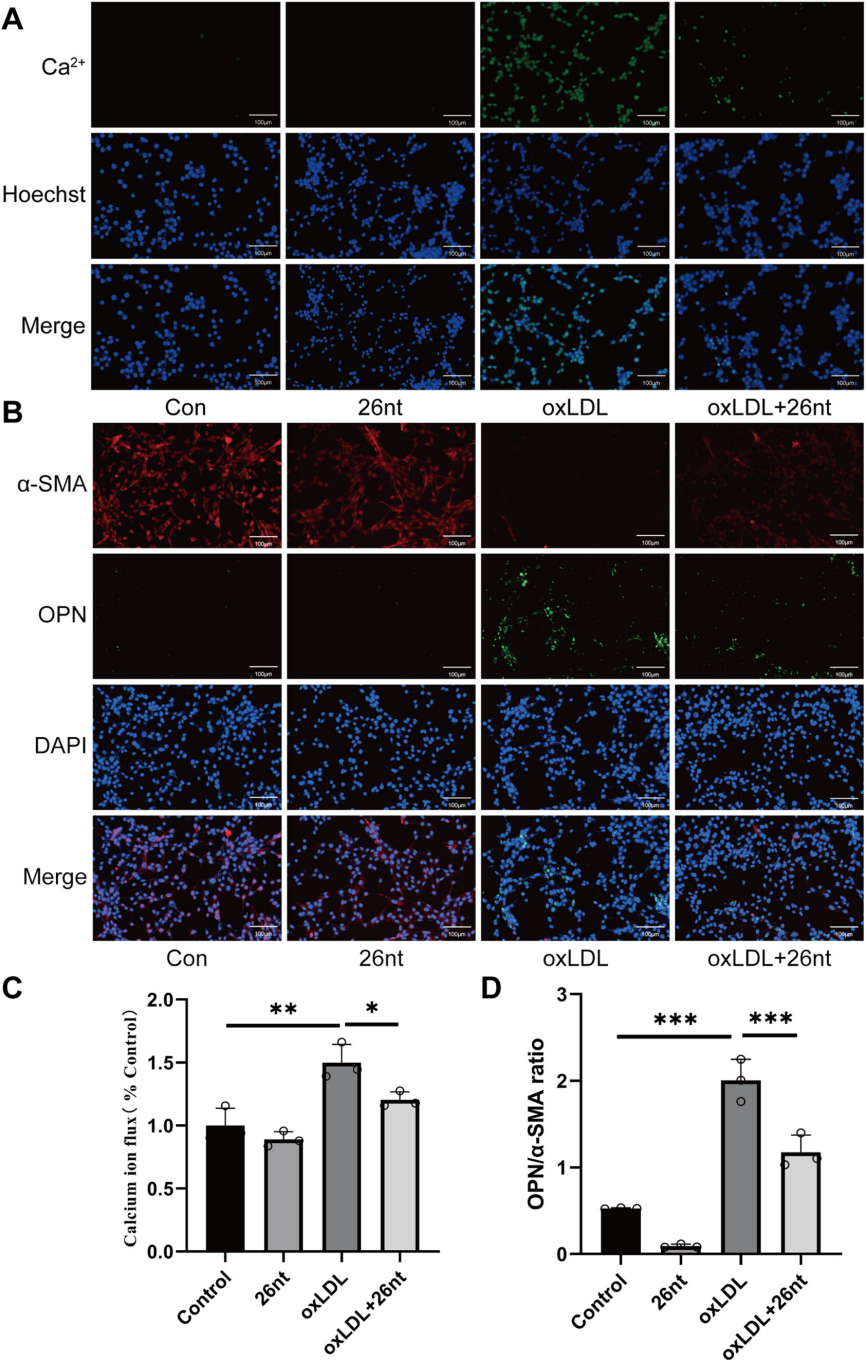
In vitro, X26nt reduces cellular calcium overload under oxLDL conditions and promotes VSMC transition from synthetic to contractile phenotype. (A, B) Representative images of calcium ion flux (A) and immunofluorescence staining for OPN and α‐SMA (B) in oxLDL‐treated VSMCs treated with control or X26nt. (C, D) Quantitative analysis of calcium ion flux and VSMC phenotype switching ratio. *n* = 3 per group. Data are expressed as mean ± SD. Statistical significance was determined using one‐way ANOVA and Tukey's post hoc test. *p < 0.05; **p < 0.01; ***p < 0.001; ns, not significant.

Excessive ROS lead to abnormal calcium ion accumulation within the cells. This study further investigated the effects of oxLDL on calcium ion overload in VSMCs, finding that oxLDL significantly promoted calcium ion overload, which could be partially inhibited by X26nt (Figure 7A,C). Moreover, ROS play a pivotal role in smooth muscle phenotypic transformation by regulating transcription factors and signaling pathways, such as MAPK and PI3K/Akt. Additionally, oxLDL induced a shift of VSMCs from the contractile to the synthetic phenotype, a process that was partially reversed by X26nt(Figure 7B,D).

## 4 Discussion

Atherosclerosis is widely recognized as a chronic immune‐inflammatory disease marked by lipid accumulation, oxidative stress, and immune cell infiltration into the vascular wall [15]. VSMCs, once viewed primarily as structural elements, are now understood to actively contribute to plaque progression through phenotypic switching in response to inflammatory stimuli [8, 16]. In this context, understanding the mechanisms that regulate VSMC behavior is crucial not only for deciphering vascular remodeling but also for identifying therapeutic targets that can modulate the inflammatory microenvironment driving atherosclerosis.

Exosomes have emerged as key mediators of intercellular communication in vascular diseases, capable of delivering diverse bioactive molecules—such as microRNAs, long noncoding RNAs, and proteins—that modulate immune responses and endothelial function [17, 18]. Under pathological conditions like atherosclerosis, the molecular cargo of exosomes can dynamically change, transmitting either harmful or protective signals depending on the cellular context [19]. For example, miR‐155‐5p–enriched exosomes have been shown to exacerbate endothelial injury and accelerate atherosclerosis progression [20], while exosomal changes induced by hypercholesterolemia further worsen vascular inflammation and myocardial damage [21]. However, not all exosomes from atherosclerosis‐related environments are detrimental. This study identified a specific subset of VSMC‐derived exosomes enriched in X26nt that confer vasculoprotective effects by reducing oxidative stress, inhibiting phenotypic switching, and maintaining endothelial integrity. These findings highlight the critical role of functional cargo characteristics—rather than simply cargo abundance—in determining the biological outcomes of extracellular vesicles. Indeed, emerging evidence suggests that even traditionally “protective” factors, such as HDL, may have paradoxical effects under pathological conditions, challenging the simplistic view that “more is better” [22].

This study reveals a novel function for exosomal X26nt, a 26‐nucleotide RNA fragment excised during IRE1α‐mediated splicing of XBP1, in modulating VSMC phenotype and redox homeostasis. Elevated X26nt expression was observed in the arterial walls of atherosclerotic mice, and functional experiments demonstrated that exosomes derived from VSMCs—enriched with X26nt—reduced oxLDL‐induced phenotypic switching and mitochondrial stress in recipient VSMCs. Synthetic X26nt treatment resulted in decreased HMGB1 and XBP1 expression, alongside increased SOD1 expression at both the transcript and protein levels, especially under pro‐atherogenic conditions. These findings suggest that X26nt may be involved in a previously unrecognized feedback loop within the ER stress response, linking splicing byproducts to immune‐metabolic regulation in vascular diseases.

Functionally, exosomal X26nt partially restored the contractile phenotype of VSMCs, as shown by increased α‐SMA and decreased OPN expression [23, 24]. In contrast, exosomes from IRE1α‐ or XBP1‐silenced VSMCs failed to produce this effect, highlighting the biological specificity of X26nt as a functional cargo. These results underscore the role of VSMC‐derived exosomes in mediating paracrine signaling during vascular inflammation and remodeling. Additionally, immunofluorescence staining of endothelial cells revealed that X26nt partially reversed oxLDL‐induced downregulation of CD31, suggesting protective effects beyond VSMCs.

At the molecular level, luciferase reporter assays confirmed that X26nt directly targets the 3’ untranslated regions (3'UTRs) of XBP1 and c‐FOS, two key mediators of ER stress and pro‐inflammatory responses [25, 26]. Suppression of these targets introduces a novel layer of posttranscriptional regulation within the IRE1α–XBP1 axis. Furthermore, the reduction of HMGB1 expression following X26nt treatment suggests broader anti‐inflammatory effects, as HMGB1 is a key damage‐associated molecular pattern (DAMP) involved in amplifying immune activation [27].

Although the SM22α promoter in our AAV2 construct is commonly used to achieve VSMC‐preferential expression, AAV2 itself lacks inherent cell‐type specificity. Consequently, limited off‐target transduction in non‐VSMC tissues cannot be completely excluded. Future studies utilizing inducible lineage‐tracing or cell‐restricted delivery strategies (e.g., Cre‐loxP systems or tissue‐tropic serotypes) will be essential to further validate the specificity and safety of X26nt‐based interventions. Additionally, the long‐term effects of X26nt expression in vivo remain to be investigated; extended treatment studies will be crucial to evaluate the durability and safety of X26nt‐based therapeutic strategies.

## 5 Conclusion

In conclusion, exosomal X26nt serves as a critical modulator of VSMC phenotypic plasticity and oxidative stress through the c‐FOS/XBP1/SOD1 axis. These findings enhance the understanding of ER stress–derived noncoding RNAs as active regulators of immune‐inflammatory processes in vascular remodeling. Targeting exosomal RNAs like X26nt offers a promising therapeutic strategy for atherosclerosis and related vascular disorders, further reinforcing the expanding evidence supporting the immunoregulatory role of extracellular vesicles in cardiovascular disease.

## Author Contributions

**Zhibin Zhang:** data curation, formal analysis, methodology, writing – original draft. **Dachang Liu:** formal analysis, methodology, visualization. **Yue Zheng:** data curation, investigation, methodology, resources, writing – original draft. **Yanwu Liu:** investigation, methodology. **Xian Cheng:** validation, visualization. **Yun Chang:** software. **Xiaoyu Liang:** writing – review and editing. **Xiaomin Hu:** project administration. **Wenqing Gao:** funding acquisition, project administration.

## Ethics Statement

This study was approved by the Ethics Committee of Nankai University (no. 2022‐SYDWLL‐000486).

## Consent

All the authors have agreed to the submission and publication of this paper.

## Conflicts of Interest

The authors declare no conflicts of interest.

## Supporting information


**Supplementary Table 1:** The specific primer of X26nt and reference. **Supplementary Table 2:** The specific primer of XBP1 promoter. **Supplementary Table 3:** Primer sequences for qRT‐PCR. Figure S1 qPCR analysis of mRNA levels of HMGB1, XBP1s, SOD1, Parkin, and LC3B in VSMCs treated with or without X26nt. Figure S2 Representative immunofluorescence images showing CD31 expression in endothelial cells treated with or without X26nt.

## Data Availability

All raw data and code are available upon request.
