# Supplementary material for: Vascular Smooth Muscle‐Secreted Exosomal X26nt Impedes Atherosclerosis Progression via the c‐FOS/XBP1/SOD1 Axis
Source: Immun Inflamm Dis. 2025 Aug 26;13(8):e70251. doi: 10.1002/iid3.70251 (PMC12378562; doi:10.1002/iid3.70251)
Supplement: Supplementary file 1 — Supplementary Table 1: The specific primer of X26nt and reference. Supplementary Table 2: The specific primer of XBP1 promoter. Supplementary Table 3: Primer sequences for qRT‐PCR. Figure S1 qPCR analysis of mRNA levels of HMGB1, XBP1s, SOD1, Parkin, and LC3B in VSMCs treated with or without X26nt. Figure S2 Representative immunofluorescence images showing CD31 expression in endothelial cells treated with or without X26nt. [file IID3-13-e70251-s001.docx]

Supplementary Table 1. The specific primer of X26nt and reference.

| X26nt | GTCGTATCCAGTGCAGGGTCCGAGGTATTCGCACTGGATACGACCAGAGG | |
| --- | --- | --- |
|  | F | R |
| U6 | TGACACGCAAATTCGTGAAGC | Uni-miR qPCR primer |

Supplementary Table 2. The specific primer of XBP1 promoter.

|  | F | R |
| --- | --- | --- |
| XBP1 promoter | CAGAGAGCAGAGACAAGCAGA | GCGGACACACACACACACCTG |

Supplementary Table 3. Primer sequences for qRT-PCR

| **Target Gene** | **Primer sequence** |
| --- | --- |
| HMGB1 | F: GGCGAGCATCCTGGCTTATC  R: GGCTGCTTGTCATCTGCTG |
| SOD1 | F: AACCAGTTGTGTTGTCAGGAC  R: CCACCATGTTTCTTAGAGTGAGG |
| PARKIN | F: TCTTCCAGTGTAACCACCGTC  R: GGCAGGGAGTAGCCAAGTT |
| LC3B | F: TTATAGAGCGATACAAGGGGGAG  R: CGCCGTCTGATTATCTTGATGAG |
| XBP1s | F: AACAGAGTAGCAGCGCAGAC  R: AGCTGGAGTTTGTGGTTCTCTT |
| β-actin | F: GGCTGTATTCCCCTCCATCG  R: CCAGTTGGTAACAATGCCATGT |


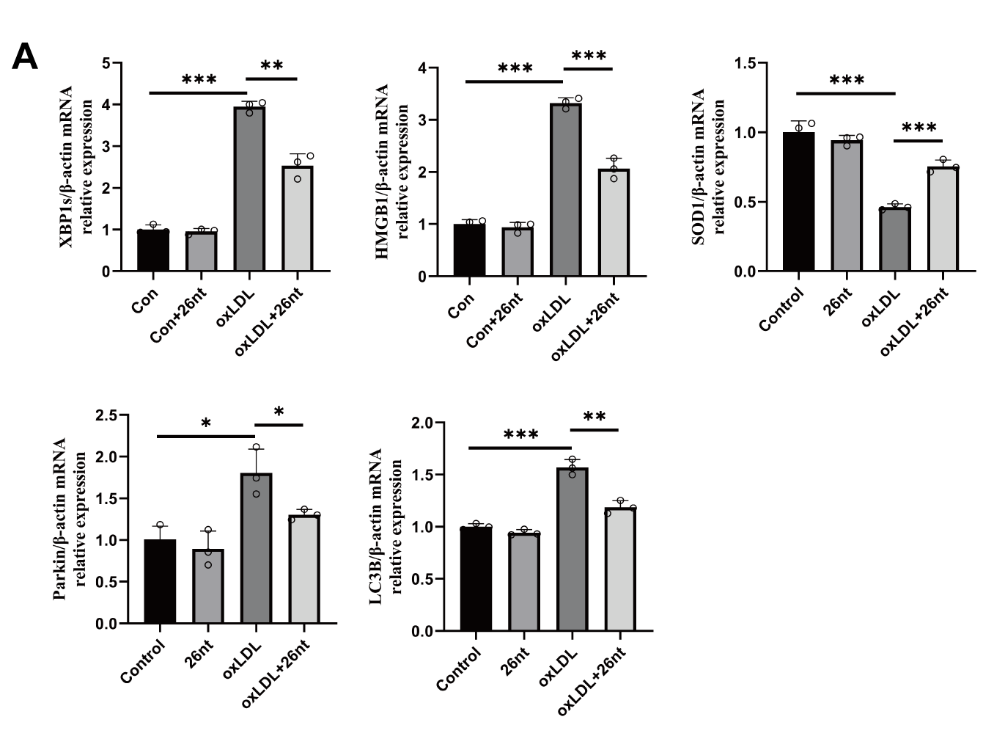


Fig. S1 qPCR analysis of mRNA levels of HMGB1, XBP1s, SOD1, Parkin, and LC3B in VSMCs treated with or without X26nt. *n* = 3 per group. Data are expressed as mean ± SD. Statistical significance was determined using one-way ANOVA and Tukey’s post hoc test. **P* < 0.05; ***P* < 0.01; ****P* < 0.001; ns, not significant.


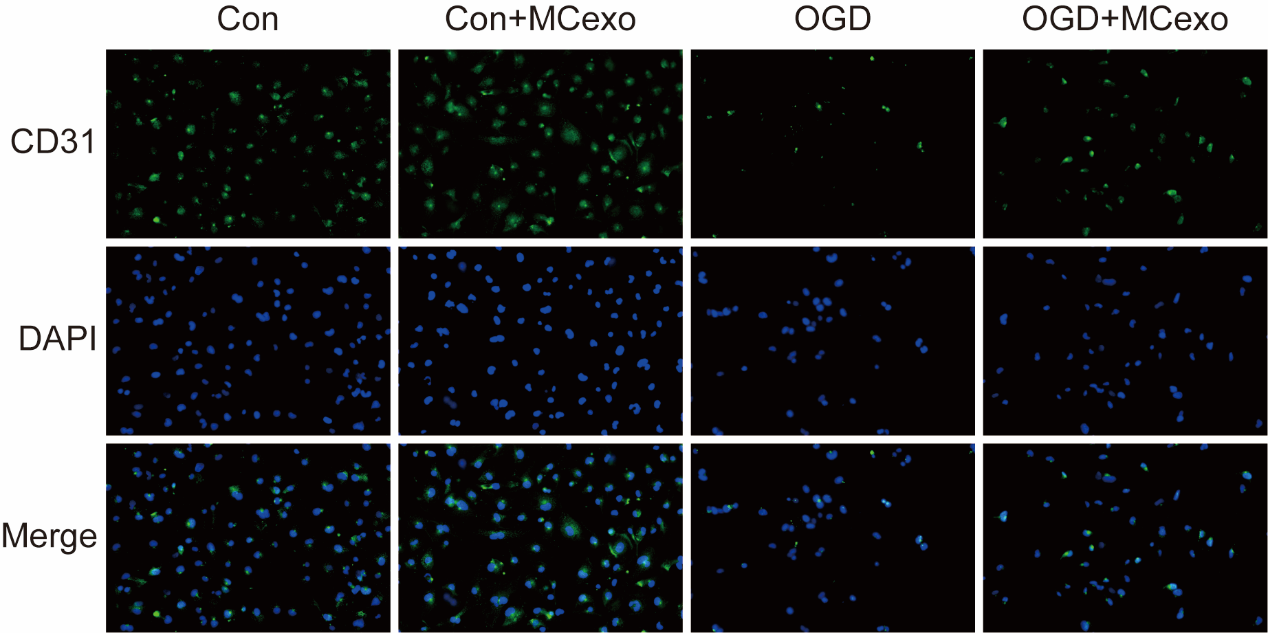


Fig. S2 Representative immunofluorescence images showing CD31 expression in endothelial cells treated with or without X26nt. *n* = 3 per group.
